# Population monitoring and conservation implications of intra‐ and interspecific nest occupation rates in swallows

**DOI:** 10.1002/ece3.70205

**Published:** 2024-10-22

**Authors:** José L. Tella, Cristina B. Sánchez‐Prieto, Pedro Romero‐Vidal, David Serrano, Guillermo Blanco

**Affiliations:** ^1^ Department of Conservation Biology Doñana Biological Station (EBD‐CSIC) Sevilla Spain; ^2^ Department of Zoology University of Granada Granada Spain; ^3^ Department of Physical, Chemical and Natural Systems Universidad Pablo de Olavide Sevilla Spain; ^4^ Department of Evolutionary Ecology Museo Nacional de Ciencias Naturales (MNCN‐CSIC) Madrid Spain

**Keywords:** conservation threats, interspecific competition, nest monitoring, nest site facilitation, nesting sites, population sizes, population trends

## Abstract

With the exception of a few groups of birds, such as large raptors and colonial seabirds, direct counts of nests cannot be conducted over very large areas for most of the abundant and widely distributed species, and thus indirect methods are used to estimate their relative abundances and population sizes. However, many species of the Family Hirundinidae (swallows and martins) build their mud nests in discrete, predictable and accessible sites, which are reused across years. Therefore, the direct count of active nests could constitute a reliable method for estimating breeding population sizes and their changes at large spatial and temporal scales. We illustrate the feasibility of this monitoring approach through a single year survey of >2700 nests of three coexisting Old‐World species, the barn swallow (*Hirundo rustica*), the red‐rumped swallow (*Cecropis daurica*), and the crag martin (*Ptyonoprogne rupestris*), distributed across Portugal and Spain. Our results revealed changes in the use of nesting substrates and increases in interspecific nest usurpation rates over recent decades. While 56% of the nests of *C. daurica* were located in rocks five decades ago, almost 100% are nowadays located in anthropogenic substrates such as bridges, road culverts, and abandoned buildings, which could have favored the range expansion of this species. Nest occupation rates were surprisingly low (12% in *C. daurica*, 21% in *H. rustica*, and 37% in *P. rupestris*), and the proportion of abandoned nesting sites was very high (65% in *C. daurica*, 50% in *H. rustica*, and 27% in *P. rupestris*). Abandonment rates reflect the population decline reported for *H. rustica*. Notably, the usurpation of nests of *C. daurica* by house sparrows *Passer domesticus,* which is the main cause of breeding failure, has increased from 2.4% in 1976–1979 to 34.7% of the nests nowadays. The long‐term monitoring of nests may constitute a reliable and affordable method, with the help of citizen science, for assessing changes in breeding population sizes and conservation threats of these and other mud‐nest building hirundines worldwide.

## Graphical abstract

The long‐term monitoring of nests may constitute a reliable and affordable method, with the help of citizen science, for assessing changes in breeding population sizes and conservation threats of mud‐nest building hirundines worldwide. Breeding adults, and completely built and destroyed nests of red‐rumped swallows *Cecropis daurica* (a1–a5), barn swallows *Hirundo rustica* (b1–b5), and rock martins *Ptyonoprogne rupestris* (c1–c5), with scales for size comparisons. Photographs: José L. Tella.

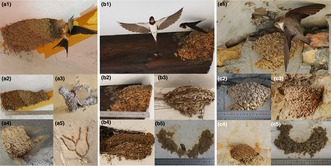

## 1 INTRODUCTION

Estimating bird population sizes and their changes over time is pivotal to assess their conservation status at regional and global scales (e.g., used as criteria for elaborating the IUCN Red List) and guide conservation actions. Moreover, bird censuses are used for assessing the effects of anthropogenic stressors on biodiversity since birds are often considered as indicators of environmental changes (e.g. Rigal et al., 2023; Stephens et al., 2016). Given the diversity of bird species and their variety of habits, life histories and abundances, a number of direct and indirect methods have been developed to monitor their populations. Direct counts allow to census breeding population sizes in a few groups of birds that are scarce or whose nests can be easily located and counted (e.g., non‐secretive large raptors, colonial herons, storks, and seabirds) (Bibby et al., 2012). However, factors such as the secretive behavior of the species, their large abundances and/or distributions make only indirect methods affordable for the vast majority of birds, including most passerine species. Indirect survey methods such as line transects and point counts allow the estimation of relative abundances that can be compared among sites and over time, or extrapolated to the species' ranges to estimate their population sizes (Bibby et al., 2012).

The family Hirundinidae (swallows and martins, hereafter hirundines) is distributed across the globe and stands out among passerines by the capacity of several species to build their own nests using mud (Turner, 2004). Mud‐nest building is a recent evolutionary trait that served as a key innovation in allowing hirundines to colonize previously unsuitable habitats, the elaboration of new social systems, and the increase of breeding densities in this clade (Winkler & Sheldon, 1993). Notably, the locations of mud‐nests are easily predictable as they are built in conspicuous sites such as cliffs, caves and buildings, and may be reused over the years (Brown & Brown, 1996; Safran, 2004; Turner, 2004). Therefore, the direct count of active nests can constitute a reliable method for estimating the population sizes (Ambrosini et al., 2012; Brown & Brown, 1996) and their changes at large spatial and temporal scales. Reliable monitoring methods are needed given the evidence of large‐scale population declines of several hirundines in North America and Europe (Michel et al., 2021; SEO/BirdLife, 2022; Woodward et al., 2018).

Here, we illustrate the potential feasibility of this nesting‐based monitoring approach through a single‐year survey of the nests of three coexisting Old‐World species, the barn swallow (*Hirundo rustica*), the red‐rumped swallow (*Cecropis daurica*), and the crag martin (*Ptyonoprogne rupestris*), across Portugal and Spain. Moreover, our baseline survey also revealed poorly known changes in the use of nesting substrates and increases in interspecific nest usurpation rates over recent decades, with conservation implications.

## 2 MATERIALS AND METHODS

During the 2023 breeding season, we sampled several areas scanning for nests of the three swallow species across most of their distribution ranges in Portugal and Spain. We randomly traveled periurban, rural and natural areas looking for potential nesting sites which could be used or not by the species. We did not attempt to perform complete local nest censuses but rather a good representation of nests located in different habitats, altitudes (from the sea level to 1500 m a.s.l.), and nesting substrates to obtain a general picture of nesting patterns at a large spatial scale. We designed this baseline survey for *H. rustica* and *C. daurica*, but also recorded the nests eventually found of the scarcer and poorly known *P. rupestris*. We did not record the nests of the fourth sympatric mud‐nest building hirundine, the house martin (*Delichon urbicum*), since it mostly breeds in the streets of urban areas and thus surveying this species would require a different design, focused on urban settlements.

We defined nesting sites as discrete sites (e.g., isolated caves, isolated buildings or bridges) where one or more nests from one or more hirundine species were found breeding solitarily or colonially. This means that a nesting site may range from a building with a single nest of one species to a building with all three species and tens of nests. To illustrate results and make comparisons with previous literature, nesting sites were grouped into natural sites (caves, rocks and small cliffs), used buildings (i.e., buildings used as homes or work places), abandoned buildings (i.e., those no longer used for daily human activities), bridges, and road culverts.

**FIGURE 1:**
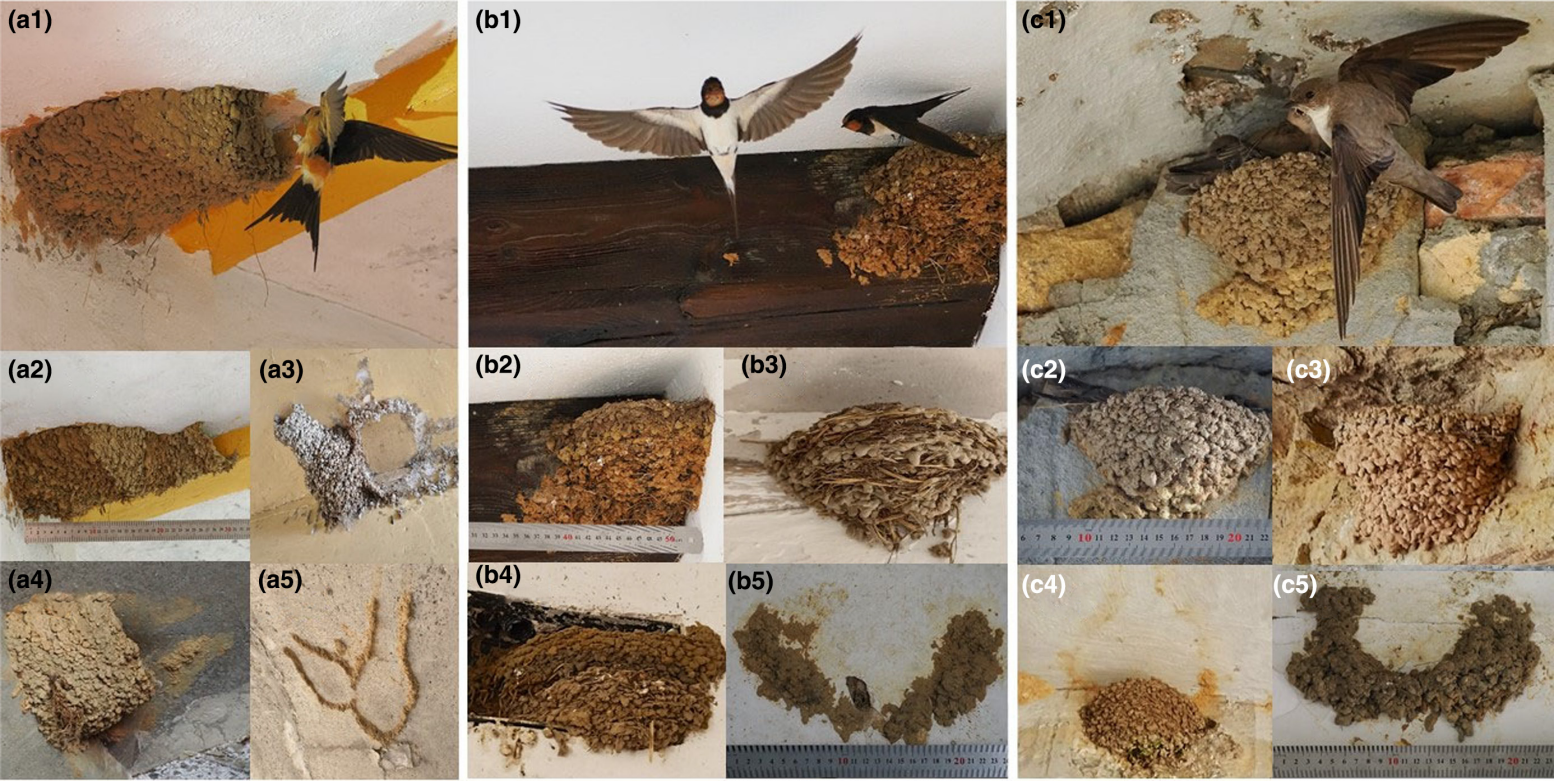
Breeding adults, and completely built and destroyed nests of red‐rumped swallows *Cecropis daurica* (a1–a5), barn swallows *Hirundo rustica* (b1–b5), and rock martins *Ptyonoprogne rupestris* (c1–c5), with scales for size comparisons. Photographs: José L. Tella.

The nests of *C. daurica* are unmistakable since they build large enclosed nests with an entrance tunnel (Figure 1a1–a5), while *H. rustica* and *P. rupestris* build much smaller open cup nests (Figure 1b1–b5,c1–c5, respectively). Nests of the latter two species could not be confounded with broken nests of *C. daurica* since the silhouette of the closed nest and its tunnel is marked with remains of mud (Figure 1a4) even when the nest collapses and is completely destroyed (Figure 1a3,a5). See for comparison a nest of *P. rupestris* built below a collapsed nest of *C. daurica* (Figure 1c4). However, the nests of *H. rustica* and *P. rupestris* are in some cases almost identical, as those we selected for illustrating Figure 1b1,b2,c1,c2. Nonetheless, there are some clues that, with experience acquired, help differentiate them. *P. rupestris* nests tend to be smaller and have a more rounded shape (Figure 1c3–c5) than *H. rustica* nests (Figure 1b3–b5). Nest location may also contribute, since when *P. rupestris* and *H. rustica* rarely coincide breeding in buildings the former usually build its nests in the external walls while the latter usually do it in the porch and inside rooms.

**FIGURE 2:**
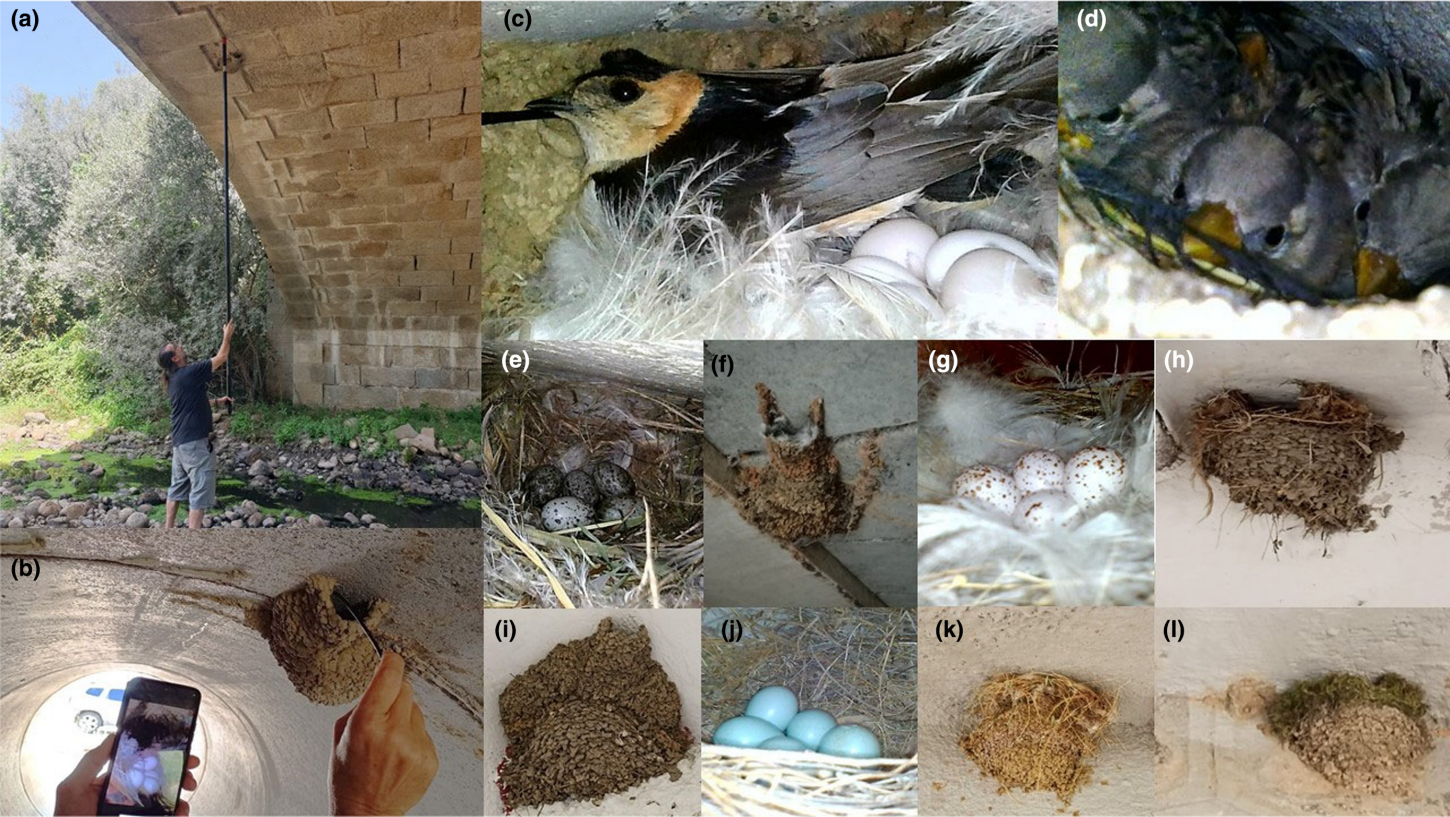
Use of an endoscope for inspecting the interior of nests of *Cecropis daurica* sited under a bridge (a) and inside a road culvert (b), showing a female incubating in a nest cup typically lined with white feathers (c). Nests of *C. daurica* usurped by rock sparrows *Petronia petronia* [(d) with fledglings], house sparrows *Passer domesticus* [(e) with eggs, note nest chamber mostly lined with grasses], and by white‐rumped swifts *Apus caffer* [(f) note the tunnel lined with white feathers until the entrance]. Nest of *H. rustica*, typically linned with feathers, with eggs (g), usurped by *P. domesticus* [(h) note the open mud cup almost covered with grasses], usurped by *C. daurica* [(i) note the nest with tunnel built on the top of the *H. rustica* nest], and by blue rock thrush *Monticola solitarius* (j). Nest of *P. rupestris* usurped by *P. domesticus* [(k) note the grass ball covering it] and by Eurasian wren *Troglodytes troglodytes* [(l) note the moss ball covering it]. Photographs: José L. Tella and Cristina B. Sánchez‐Prieto.

We surveyed nesting sites between April and August, when the breeding season of the three species overlaps, with one to three nesting attempts per pair (de Lope, 1981b). Given the latitudinal range of our study area, the northern populations initiated the reproduction about 3 weeks later than southern populations. During visits to nesting sites, the status of each nest was recorded as destroyed (when some mud remained attached to a nesting substrate, indicating there was a completely built nest in the past, Figure 1a5,b5,c5), unoccupied (partially broken or completely built nests, that could be used for nesting but were not used), or occupied by the nest‐building species or by other species. Nest occupation was determined by the observation of nest contents including eggs or chicks and/or the presence of adults building the nests, incubating or feeding chicks. Occupied nesting sites were visited 2–8 times for obtaining detailed information on breeding parameters for a parallel, ongoing study (results not shown here). Nesting sites with unoccupied nests were revisited at least once at the end of the breeding season to ensure their occupation status. The occupied nests of swallows were easily recognized as they lined their nest chambers with feathers, mostly silky white ones. White‐rumped swifts (*Apus caffer*) usurping nests of *C. daurica* also used white feathers, but lined the whole nest until the entrance of the tunnel. Other species such as sparrows (*Passer* sp.) used large amounts of green and dried grasses, or green mosses in the case of Eurasian wrens (*Troglodytes troglodytes*) (see Figure 2 for a variety of nest occupation examples). While most of the open cup nests of *H. rustica* and *P. rupestris* were easily inspected from a distance with binoculars or using ladders, the enclosed nests of *C. daurica* made difficult its inspection. Then, we used a Pancellent Mini USB HD Camera endoscope (8 mm lens diameter) attached with a 5 m cable to the screen of a smartphone, and elevated with telescopic poles when necessary, for inspecting nest chambers of *C. daurica* and inaccessible nests of the other two species (Figure 2a,b). Thus, we were able to systematically record the occupancy of nests both by the owner and usurping species.

## 3 RESULTS

### 3.1 Nesting sites

**FIGURE 3:**
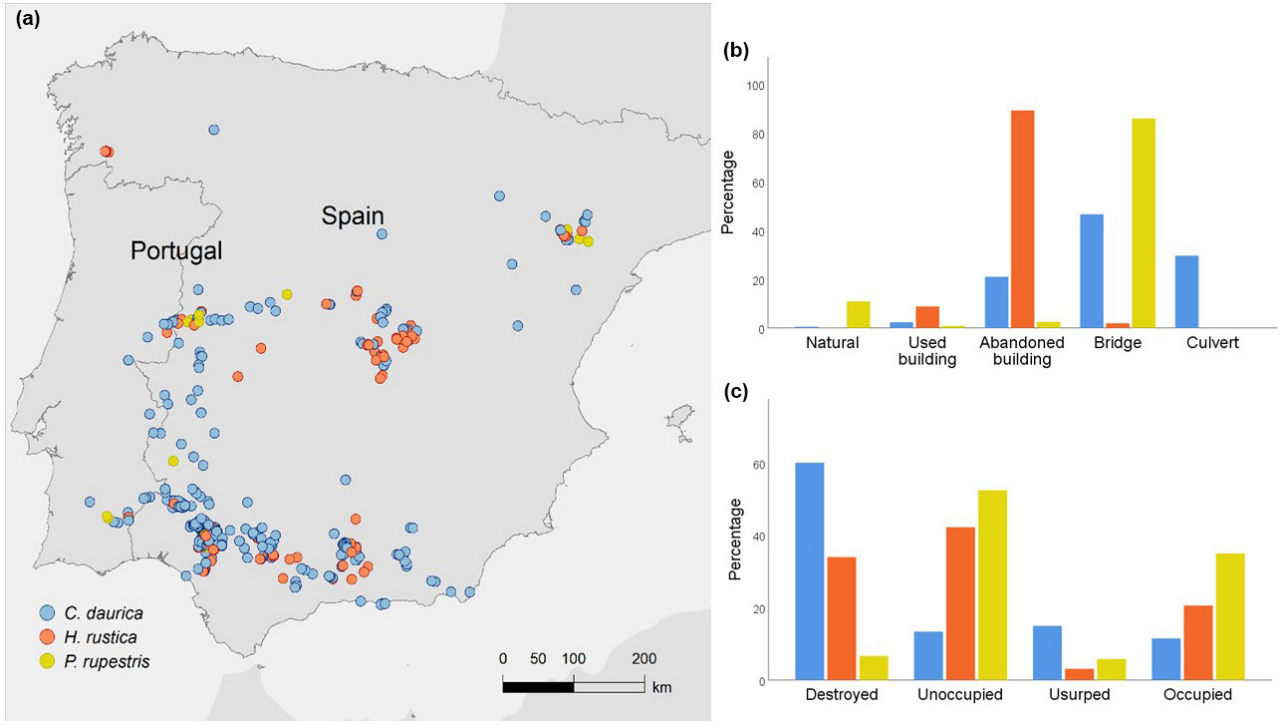
(a) Sites (*n* = 590) where nests of the three hirundine species (*n* = 2732) were located across Portugal and Spain in 2023, (b) proportion of nests of each species (same color codes) found in different nesting substrates, and (c) proportion of nests destroyed, unoccupied, occupied by the same species or usurped by other species.

We recorded 2732 nests of swallows (1389 of *C. daurica*, 1223 of *H. rustica*, and 120 of *P. rupestris*) in 590 nesting sites across Portugal and Spain in 2023 (Figure 3a). In some cases, 2–3 species coincided in the same nesting sites. Therefore, the total number of nesting sites per species reached 634 (433 for *C. daurica*, 157 for *H. rustica*, and 44 for *P. rupestris*). The distribution of nests among nesting sites differed significantly among the three species (*χ* ^2^ = 1879.69, *p* < .001, Figure 2b). While 10.83% of nests of *P. rupestris* were found in natural sites (caves and small cliffs), the use of these substrates by *C. daurica* and *H. rustica* was anecdotal (0.43% and 0.08%, respectively). Most nests of *H. rustica* were found in abandoned buildings (89.13%), followed by used buildings (8.75%). The use of abandoned and used buildings by *C. daurica* was much lower (20.95% and 2.30%, respectively). Most nests of *C. daurica* (48.74%) and *P. rupestris* (85.83%) were sited under bridges. Notably, 27.57% of *C. daurica* nests were found inside road culverts.

### 3.2 Intraspecific nest occupation

Although the use status of nests significantly varied among species (Chi‐square test, *χ* ^2^ = 518.61, *p* < .001, Figure 3c), there is a general pattern showing that most nests were destroyed or unoccupied and just a small fraction were occupied by the nest‐building species (11.52% by *C. daurica*, 20.61% by *H. rustica*, and 36.67% by *P. rupestris*).

The number of occupied nests per occupied nesting site varied among species (Kruskal–Wallis, *H* = 48.86, df = 2, *p* < .001). In the case of *C. daurica*, there was a single occupied nest in all but one case (*n* = 151), corresponding to a very large, multi‐space abandoned building where we found four occupied nests in very distant rooms. However, the number of occupied nests per nesting site ranged between 1 and 5 (mean 1.44, *n* = 32) in *P. rupestris* and between 1 and 57 (mean = 4.08, *n* = 79) in *H. rustica*.

**FIGURE 4:**
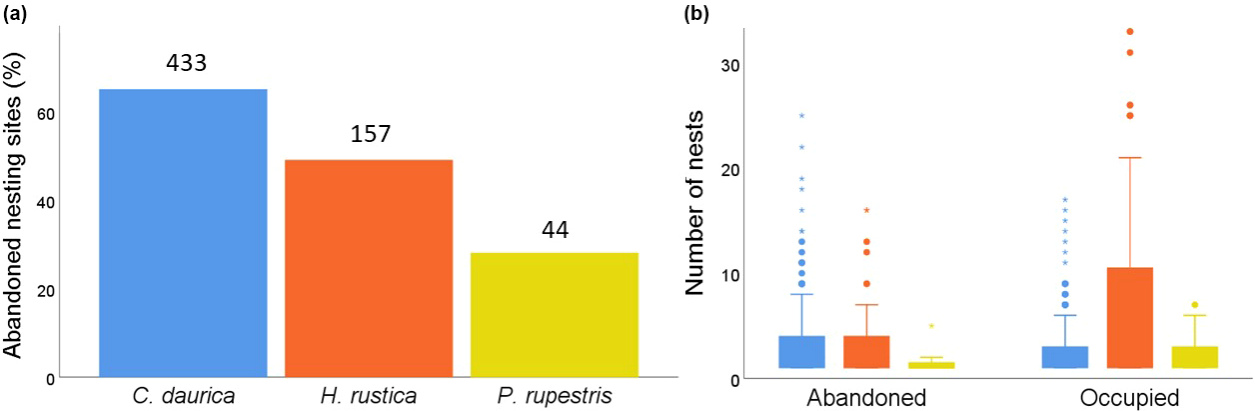
(a) Proportion of nesting sites abandoned by each swallow species, with sample sizes above bars, and (b) number of nests recorded in abandoned and occupied nesting sites of each species (same color codes).

The proportion of abandoned nesting sites significantly varied among species (*χ* ^2^ = 30.56, *p* < .001, Figure 4a), being much larger in *C. daurica* (65.13%) than in *H. rustica* (49.68%) and *P. rupestris* (27.27%). The total number of nests recorded did not differ between abandoned and occupied nesting sites of *C. daurica* (Mann–Whitney *U* test, *Z* = −1.169, *p* = .243), but was smaller in abandoned than that in the occupied ones of *H. rustica* (*Z* = −2.732, *p* = .006) and *P. rupestris* (*Z* = −2.327, *p* = .028) (Figure 4b).

### 3.3 Interspecific nest occupation

Swallow nests were usurped by up to 11 species of birds and, anecdotally, by potter wasps and snails that obstructed the nests of *C. daurica* (Table 1). The percentage of nests occupied by other species was small in *H. rustica* (3.11%) and *P. rupestris* (5.83%), but was even larger (14.97%) than the proportion of used by the owner species (11.52%) in the case of *C. daurica* (Figure 3c). When only considering the potentially available nests (i.e., excluding destroyed nests) the percentage of nests usurped by other species remained low in *H. rustica* (4.71%) and *P. rupestris* (7.11%) but increased to 37.55% in *C. daurica* (Table 1). The house sparrow (*Passer domesticus*) was the most common usurping species, occupying up to 92.31% of the usurped nests and 34.66% of all the available nests (*n* = 554) in the case of *C. daurica*.

**TABLE 1:** Interspecific occupation of nests of the three swallow species studied, showing the number of nests usurped (*n*), the percentage over the total of usurped nests (%UN), and the percentage over all the available nests, i.e., excluding those destroyed (%AN).

Usurping species	Host species								
	*Cecropis daurica*			*Hirundo rustica*			*Ptyonoprogne rupestris*		
	*n*	%UN	%AN	*n*	%UN	%AN	*n*	%UN	%AN
Birds									
White‐rumped swift *Apus caffer*	4	1.92	0.72						
Red‐rumped swallow *Cecropis daurica*				4	10.53	0.50	1	14.29	0.89
Barn swallow *Hirundo rustica*	1	0.48	0.18						
Blue rock thrush *Monticola solitarius*				1	2.63	0.12			
Pied wagtail *Motacilla alba*							1	14.29	0.89
Black wheatear *Oenanthe leucura*				1	2.63	0.12			
House sparrow *Passer domesticus*	192	92.31	34.66	29	76.32	3.59	2	28.57	1.79
Spanish sparrow *Passer hispaniolensis*	1	0.48	0.18	2	5.26	0.25			
Rock sparrow *Petronia petronia*	1	0.48	0.18						
Black redstar *Phoenicurus ochruros*				1	2.63	0.12			
Eurasian wren *Troglodytes troglodytes*	5	2.40	0.90				3	42.86	2.68
Invertebrates									
Potter wasps *Sceliphron destillatorium*	3	1.44	0.54						
Snails *Cornu aspersum*	1	0.48	0.18						
Total	208		37.55	38		4.71	7		7.11

## 4 DISCUSSION

Here we show how the nesting habits of mud‐nest building hirundines that cluster their nests at discrete and predictable sites, which can be reused over decades, may allow the direct monitoring of their breeding populations sizes at different spatial and temporal scales. Moreover, our single‐year baseline survey offers insights on some poorly known aspects of their natural histories such as potential changes in nesting sites, population sizes, and interspecific competition for nests, as discussed below.

While *H. rustica* has become a model species for scientific research and is considered as one of the world's best‐known birds, knowledge on the natural history of *C. daurica* and *P. rupestris* is poor (Turner, 2004). *C. da*urica is supposed to have colonized the Iberian Peninsula, through Southern Spain and Portugal, in the early decades of the 20th century, then slowly spreading across almost the whole Peninsula and Southern France (de Lope, 1981a). However, most information on its natural history is limited to the extensive work conducted by de Lope (1981b) between 1976 and 1977. At that time, 56% of the nests (*n* = 589) were located in rocks while only 44% were in bridges (de Lope, 1981a). Surprisingly, our survey conducted almost five decades later showed that <1% of nests are located in natural substrates, while most of the nests are nowadays in bridges, road culverts, and abandoned buildings. This change in nesting site selection is consistent with the innovation advantage acquired by mud‐nest building hirundines, allowing them to colonize otherwise unsuitable habitats (Winkler & Sheldon, 1993), and supports the hypothesis that the increased use of human constructions could have allowed the large‐scale spread of the species (de Lope, 1981a). In fact, the use of abandoned buildings and road culverts seems to have also favored the species in very distant areas of its world distribution, such as Albania (Fasola et al., 1997) and India (Ali et al., 2022). The large proportion of nests of *P. rupestris* located in bridges—and even buildings—is also relevant for a species that is considered more linked to natural substrates (Turner, 2004). This use of anthropogenic sites is surely overestimated since, as explained above, our surveys were designed for *C. daurica* and *H. rustica*. Therefore, the surveyed areas did not include large cliffs where, through our work on other cliff‐nesting species, we know *P. rupestris*—but not *C. daurica*—nests frequently. Nonetheless, *P. rupestris* could be experiencing the same nesting‐substrate switching behavior experienced in the past by the other three sympatric species (including *D. urbicum*) to finally nest almost exclusively in anthropogenic sites, which merits a further long‐term assessment.

Another surprising result is the low percentage of nests occupied, ranging from 37% in *P. rupestris* to only 11% in *C. daurica*. Some nests are destroyed when they collapse between few days and up to 8 years after construction, while others remain intact for more than 10 years (J. L. Tella, C. B. Sanchez‐Prieto, P. Romero‐Vidal, D. Serrano and G. Blanco, unpublished observations). Nest collapse is probably related to the low plasticity of some of the muds used by swallows (Papoulis et al., 2018) which could not support the weight of their nests, especially in the case of the large nests of *C. daurica*, the species for which we found 60% of the nests destroyed. Nonetheless, the pairs may rapidly build new nests (within 2 weeks, J. L. Tella and C. B. Sanchez‐Prieto, unpublished observations; de Lope, 1981b) if there is available mud in the surroundings. These observations, together with the presence of a high proportion of available but unoccupied nests and nesting sites with only old nests, suggests there is a decline in breeding population numbers. However, we are unable to know how many breeding pairs were lost based on these unoccupied nests, which could only be assessed by repeating this survey over years. Nonetheless, the percentage of abandoned nesting sites could be considered as a qualitative indicator of population decline. We know the species disappeared as breeder in a number of nesting sites, although we do not know how many pairs disappeared. Abandoned nesting sites held lower numbers of nests than occupied ones in the case of *H. rustica* and *P. rupestris*, which is in agreement with the longer persistence of large colonies in other mud‐nest building hirundines (Brown & Brown, 1996) and with the preference of individuals of *H. rustica* to breed in sites with a larger availability of old nests (Safran, 2004). Consistently, there is no difference in the number of nests between abandoned and occupied nesting sites of *C. daurica*, which mostly breeds solitarily (de Lope, 1981b; Turner, 2004).

Repeated surveys of nesting sites over years are needed to ascertain to what extent the high abandonment rates may be related to stochasticity or particularities of the single‐year surveyed, or to long‐term population declines and differences in the population dynamics among the studied species. Notably, the percentage of abandoned nesting sites in the case of *H. rustica* (49.7%) match well with the population decline of the species estimated in Spain between 1998 and 2018 (−51.1%) through indirect methods (relative abundances obtained from line transects, de Lope, 2022). Based on this information, the species has been cataloged as Vulnerable in the Spanish Red List of Birds (López‐Jiménez, 2021). However, the high percentage of abandoned nesting sites of *C. daurica* (65.1%) greatly contrasts with the population stability or even a slight population increase estimated through the same methodology (de Lope, 2022). Nonetheless, BirdLife International (2024) claims that population sizes of *C. daurica* within Europe are poorly known and that monitoring programmes should be implemented across its range to more accurately determine its status. The reliability of population size estimates predicted through indirect sampling at large spatial scales and inference from statistical procedures need to be tested through comparison with direct counts conducted in selected plots (Blanco et al., 2012, 2014), which would result easy in the case of the mud‐nest building hirundines we surveyed.

A third surprising result of our survey is related to the interspecific occupation of nests. As in the case of other nest‐facilitator species (e.g., Hernández‐Brito et al., 2021), the mud‐nests of hirundines may persist for years and be used by other species for nesting (Turner, 2004). While the percentage of nests of *H. rustica* and *P. rupestris* occupied by other species is relatively low, in agreement with previous information (de Lope, 1981b), this percentage is even higher than the percentage of nests used by the owner species in the case of *C. daurica*. Moreover, it has drastically increased in recent decades. In 1976–1979, de Lope (1981c) found only 6.4% of *C. daurica* nests occupied by other bird species after surveying a large number of nests (*n* = 594) across most of the range distribution of the species in Spain and Portugal at that time. Notably, the occupation by *P. domesticus* increased from 2.4% (de Lope, 1981c) to 34.7% of the available nests nowadays. Moreover, when restricting our data to the same provinces surveyed by de Lope (1981c) for a better comparison, the usurpation of available nests by *P. domesticus* increases up to 53.82% (*n* = 314). This species not only occupies nests abandoned by the swallows but also evicts them from occupied ones, expelling adult swallows, their eggs and small chicks (J. L. Tella and C. B. Sanchez‐Prieto, personal observation; de Lope, 1981c; Turner, 2004). In fact, we found nest usurpation by *P. domesticus* caused total breeding failure in 26.37% of the *C. daurica* nests monitored in 2023 (*n* = 91), being the main cause of unsuccessful breeding (J. L. Tella, C. B. Sanchez‐Prieto, P. Romero‐Vidal and D. Serrano, unpublished data). These observations confirm that the species may be threatened by competition with other birds which use its nests (BirdLife International, 2024). Paradoxically, nest facilitation by *C. daurica* has positive conservation counterparts for other species. It allows *P. domesticus* to breed in sites far from human settlements that otherwise would be unsuitable for the species, which is declining in Spain (Murgui, 2022), and the colonization from sub‐Saharan quarters and further range spread of *A. caffer*, which uses almost exclusively the nests of *C. daurica* for breeding (Prieta Díaz, 2022).

Concluding, we have shown through a simple survey the feasibility of monitoring mud nests and nesting sites of hirundines at large spatial scales. Swallows and martins are friendly and charismatic for society and nest in accessible places, so people could easily be involved in citizen science programs. In fact, citizen science surveys, recruiting a number of volunteers, have already been developed to record the breeding performance of hirundines at large spatial (Imlay et al., 2018) and even national scales (Kettel et al., 2021). Therefore, a large number of small representative plots could be selected, covering the distribution of the species well, where all the nests present, their occupancy status, and rebuilding of destroyed nests could be recorded. Within these selected plots, all sites suitable for nesting—not only those with known prior nesting—should be surveyed, thus allowing the detection of the colonization of new sites and nesting‐site turnover. The breeding densities obtained from these plots can be extrapolated to the areas occupied by the species to obtain estimates of population sizes at regional or national scales in a single year. The long‐term monitoring of these plots would offer estimates of spatial and temporal changes in population sizes based on direct counts, could allow testing the reliability of estimates based on indirect methods, and offer valuable information on changes in the natural history, interspecific competition and conservation threats of the species. This approach, not new for a selected group of species whose nests are easily counted (Bibby et al., 2012), can be extended to a number of mud‐nest building hirundines that are distributed across the world (Turner, 2004). Moreover, the repeated monitoring of a good sample of nests throughout the breeding season could help to disentangle whether population dynamics are related to changes in breeding phenology and performance (Imlay et al., 2018).

## AUTHOR CONTRIBUTIONS

**José L. Tella:** Conceptualization (equal); data curation (equal); formal analysis (equal); investigation (equal); methodology (equal); writing – original draft (lead); writing – review and editing (equal). **Cristina B. Sánchez‐Prieto:** Conceptualization (equal); data curation (equal); formal analysis (supporting); investigation (equal); methodology (equal); writing – review and editing (equal). **Pedro Romero‐Vidal:** Investigation (supporting); writing – review and editing (equal). **David Serrano:** Investigation (supporting); writing – review and editing (equal). **Guillermo Blanco:** Investigation (supporting); writing – review and editing (equal).

## FUNDING INFORMATION

CBS‐P was supported by projects TED2021‐132283B‐100 of Spanish Ministry of Science and Innovation and P21 00118 (MEDAVES) from Junta de Andalucia, and PR‐V by a Margarita Salas Grant (NextGenerationEU, by the Recovery, Transformation and Resilience Plan and by the Ministry of Universities for the Requalification of the Spanish University system 2021–2023, convened by the Pablo de Olavide University, Seville). Fieldwork was conducted without financial support.

## CONFLICT OF INTEREST STATEMENT

The authors declare that there are no conflicts of interest.

## Data Availability

The data that supports the findings of this study are available in Table 1 and Section 3 included in the article. The location of all the nests is available under request to authors.
